# Clinicopathological Analysis of the ISUP Grade Group And Other Parameters in Prostate Cancer: Elucidation of Mutual Impact of the Various Parameters

**DOI:** 10.3389/fonc.2021.695251

**Published:** 2021-07-28

**Authors:** Yoichiro Okubo, Shinya Sato, Kimito Osaka, Yayoi Yamamoto, Takahisa Suzuki, Arika Ida, Emi Yoshioka, Masaki Suzuki, Kota Washimi, Tomoyuki Yokose, Takeshi Kishida, Yohei Miyagi

**Affiliations:** ^1^Department of Pathology, Kanagawa Cancer Center, Kanagawa, Japan; ^2^Molecular Pathology and Genetics Division, Kanagawa Cancer Center Research Institute, Kanagawa, Japan; ^3^Department of Urology, Kanagawa Cancer Center, Kanagawa, Japan; ^4^Department of Radiology, Kanagawa Cancer Center, Kanagawa, Japan; ^5^Department of Pathology, University of Tokyo Hospital, Tokyo, Japan

**Keywords:** prostate, grade group, Gleason Score, metastasis, adenocarcinoma, lymphatic invasion, biochemical recurrence

## Abstract

**Background:**

Prostate cancer has become increasingly common worldwide. Although Grade group (GG) is widely accepted as an indicator of prostate cancer grade, there are malignancies that cannot be defined by GG alone. Moreover, the relationship between GG and other parameters remains unclear. Herein, we aimed to explore the biological characteristics of prostate cancer.

**Methods:**

This study included 299 radical prostatectomy cases. The Chi-square test and analysis of variance were used to analyze the association of GG with binary and continuous variables. We then conducted morphological analyses. Multivariate analyses were performed to extract the data on risk factors for biochemical recurrence (BCR) and lymph node metastasis.

**Results:**

The lymphatic, venous, perineural, and seminal vesicle invasion rates were 37/299 (12.4%), 25/299 (8.4%), 280/299 (93.6%), and 23/299 (7.7%), respectively. The extraprostatic extension (EPE), positive surgical margin, tertiary Gleason pattern 5, intraductal carcinoma of the prostate gland, and lymph node metastasis rates were 89/299 (29.8%), 106/299 (35.5%), 33/260 (12.7%), 56/299 (18.7%), and 23/299 (7.7%), respectively. As GG increased, various parameters became easier to visualize; however, there were differences between the parameters. Postoperative BCR was observed in 31/242 (12.8%) cases without preoperative hormone therapy; GG2, GG3, GG4, and GG5 accounted for 4, 7, 7, and 13 cases, respectively. Multivariate analyses revealed that GG and tumor diameter were significant risk factors for early BCR, whereas lymphatic invasion, EPE, and seminal vesicle invasion were significant risk factors for lymph node metastasis. For BCR, the odds ratios (ORs) for GG and tumor diameter were 2.253 (95% confidence interval (CI]): 1.297–3.912; P=0.004) and 1.074 (95% CI: 1.011–1.142; P=0.022), respectively. For lymph node metastasis, ORs for the presence of lymphatic invasion, EPE, and seminal vesicle invasion were 7.425 (95% CI: 1.688–22.583; P=0.004), 4.391 (95% CI: 1.037–18.589; P=0.044), and 5.755 (95% CI: 1.308–25.316; P=0.021), respectively.

**Conclusions:**

We summarized various parameters correlating with each GG. Through multivariate analyses, we established the independent risk factors for early BCR and lymph node metastasis. In addition to GG, other important indices of malignancy were determined and weighted to provide a basis for future investigations.

## Introduction

Prostate cancer has become increasingly prevalent worldwide (1–4). Although the incidence rate of this tumor is lower in Japan than that in Western countries (the incidence rates in Japan, the United States, and the United Kingdom are 27.0, 98.2, and 73.2 per 100,000 population, respectively) (5), its incidence is rapidly increasing with the westernization of lifestyles (6). Most malignant prostatic neoplasms (~90%) are adenocarcinomas (7–9). In patients who are required to undergo radical prostatectomy, various parameters can be evaluated through preoperative clinical investigations and histopathological analyses of surgical specimens. These parameters include age, preoperative serum prostate-specific antigen (PSA) concentration, body mass index (BMI), tumor diameter, Grade group (GG) and Gleason score (GS), lymphatic, venous, perineural, and seminal vesicle invasion, extraprostatic extension (EPE) of the tumor, positive surgical margins, and lymph node metastasis (10). In addition, postoperative follow-up surveys allow examination of the relationship between biochemical recurrence (BCR) and various parameters after radical prostatectomy.

Among these parameters, GG (2), lymphovascular invasion (11), EPE, seminal vesicle invasion, and lymph node metastasis (12) have been established as independent poor prognostic factors. More recently, tertiary Gleason pattern 5 and intraductal carcinoma of the prostate gland (IDC-P) have also been reported as poor prognostic factors (3, 13). However, few studies have investigated lymphatic invasion and venous invasion separately (14, 15), and the relationship between GG and various clinicopathological evaluation parameters has not yet been fully elucidated. Furthermore, the extent to which each evaluation parameter affects lymph node metastasis, which is an important prognostic factor in patients with prostate cancer, remains unclear (15).

Recently, the use of robot-assisted radical prostatectomy (RARP) has gained popularity. Studies have found that RARP allows for both safe operation and efficient lymph node evaluation (16, 17). Nevertheless, one study (18) suggested that lymph node dissection using RARP does not directly contribute to the prognosis and may increase complications; however, this finding remains controversial. Therefore, in this study, instead of performing a literature search, we aimed to analyze the risk factors for lymph node metastasis using detailed morphological, immunohistochemical, and statistical analyses of surgical specimens of patients who had undergone RARP. Specifically, we initially investigated the relationship between GG and the evaluation parameters. Thereafter, we conducted a multivariate logistic regression analysis to determine the risk factors for lymph node metastasis, which has been strongly established as a poor prognostic factor postoperatively (12). We also confirmed the status of BCR after RARP, extracted risk factors using multivariate logistic regression analysis, and attempted to integrate the results with morphological analysis.

## Materials and Methods

### Identification of the Cases Used in the Analysis (Kanagawa Cancer Center, Japan)

RARP, using the da Vinci surgical system (Intuitive Surgical, Inc.; Sunnyvale, CA, United States), was introduced at our institution in August 2018. Considering the combined experience of the operators and co-medicals, prostate cancer cases treated using RARP between January 2019 and December 2020 were included in this study. In addition, for enabling the safe and most appropriate treatment using RARP, an author of this manuscript, KO, was assigned to our institution in April 2018. KO had more than four years of prior experience in operating da Vinci surgical system and had experienced approximately 400 cases before this assignment, of which he was the primary surgeon in approximately 100 cases.

Specifically, we recorded various parameters using hematoxylin and eosin (HE) staining and immunohistochemical analysis under a light microscope as our routine diagnostic procedures. In addition, a pathological diagnosis support software (“EXpath” Laboratory Information Systems for Pathology, INTEC Inc., Tokyo, Japan) was used to confirm the pathological diagnoses and clinical information. This study was performed in alignment with the tenets of the Declaration of Helsinki and approved by the Ethics Review Committee of the Kanagawa Cancer Center (Approval Number: 2019-36). Furthermore, written informed consent was obtained from the patients for the future use of their materials for research.

### Clinicopathological Parameters of the Prostate Adenocarcinoma Cases

We extracted the below mentioned clinicopathological parameters for analysis. Most of these parameters were recorded during the routine pathological diagnosis process in our institute. We also checked the medical records in May 2021 to confirm the presence of BCR. The specific tabulation method for each parameter was as follows:

#### GG

In this analysis, we adopted the 2014 International Society of Urological Pathology (ISUP) grading system for GG evaluation (19, 20). According to the invasive pattern of prostate cancer, the GG system was divided into the following five groups: GG1, 2, 3, 4, and 5 (GS: 3 + 3 = 6, 3 + 4 = 7, 4 + 3 = 7, 4 + 4 = 8, and 4 + 5 or more, respectively). We have adopted the highest GG for cases with multiple lesions. At least two pathologists evaluated the post-RARP specimens as per the 2014 ISUP system. After one of the two pathologists (YO or SS) described the primary pathology findings, the specimens were reviewed by the third pathologist (YM) using a multi-viewing biological microscope. In case of disagreement on various diagnostic findings, the three pathologists discussed; however, if they still could not agree, the opinion of the third pathologist with the longer experience as a prostate cancer diagnostician was prioritized.

#### Age

We recorded the patients’ ages when the surgery was performed.

#### BMI

BMI was determined using the patient’s body weight and height at the time of the surgery and was calculated as follows: body weight (kilograms)/height squared (meters^2^).

#### PSA Value

Each patient’s highest PSA value from the collection of the preoperative serum PSA values was recorded.

#### Tumor Diameter

After formalin fixation, we recorded the length of the prostate in three directions (vertical, transverse, and sagittal). After photography, both the prostate apex and base were examined using the cone method with sagittal sectioning (21). The remaining prostate was entirely cut at approximately 5-mm intervals from the apex to the base, perpendicular to the long axis. All sections were embedded into paraffin and examined. The pathologist examined the specimen and measured the tumor diameter. Appropriate mapping was conducted, and even the lesions in the different sections were included in the tumor diameter if they were determined to be a series of lesions based on their location. In the case of multiple lesions, the tumor diameter with the highest GG was included in this study.

#### Lymphatic and Venous Invasion

**Figure 1:**
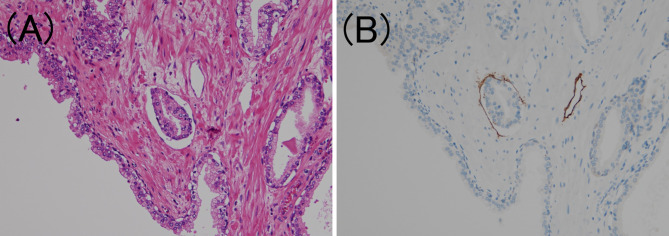
Lymphatic invasion in prostate cancer. **(A)** Small clusters of carcinoma cells are present in the lumen (hematoxylin and eosin staining, ×200). **(B)** The luminal surface of the duct is lined with D2-40 positive lymphatic endothelium (D2-40 immunohistochemistry, ×200).

**Figure 2:**
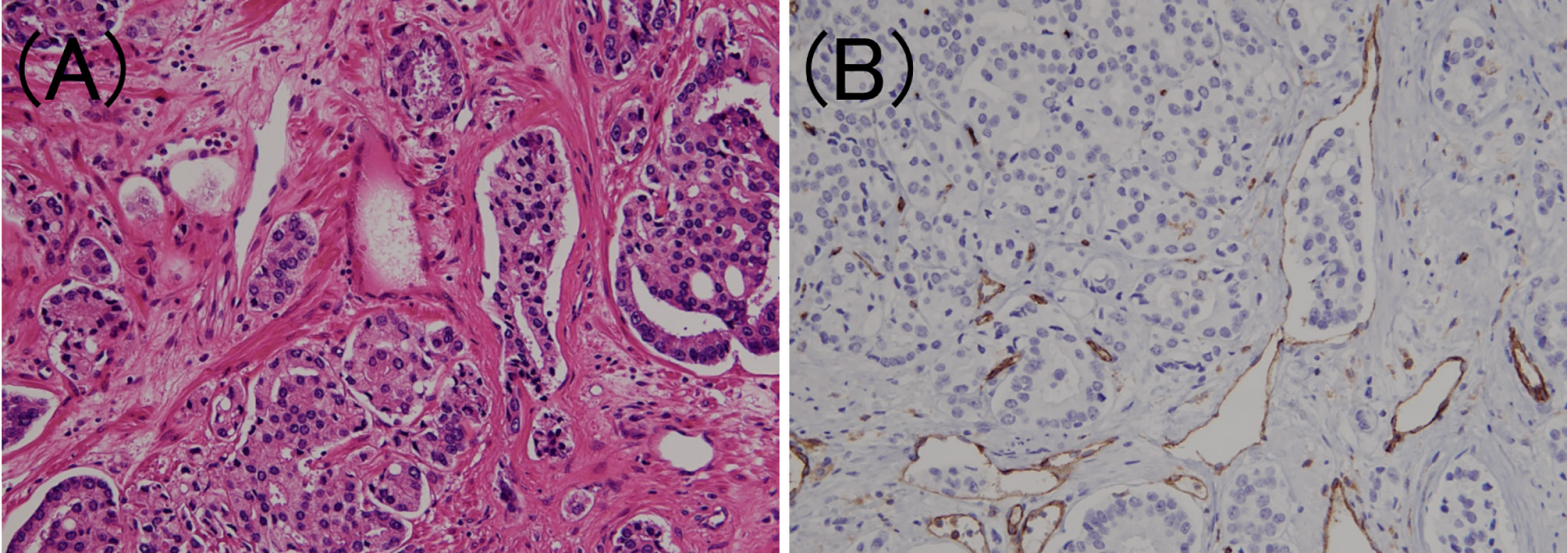
Venous invasion in prostate cancer. **(A)** Small clusters of carcinoma cells are present in the lumen (hematoxylin and eosin staining, ×200). **(B)** The luminal surface of the duct is lined with CD31 positive venous endothelium (CD31 immunohistochemistry, ×200).

**Figure 3:**
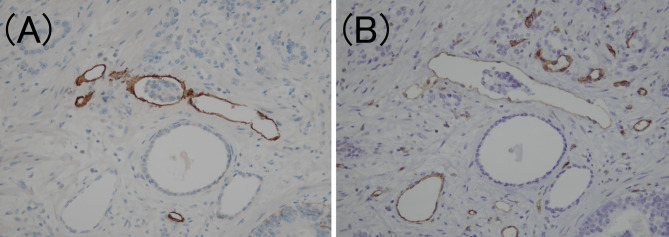
Criteria for determining lymphovascular invasion using D2-40 and CD31 immunostaining. **(A)** The lymphatic vessel is clearly stained using D2-40 immunostaining (D2-40 immunohistochemistry, ×200). **(B)** The same location as **(A)**; however, the CD31 immunostaining also faintly stains the lymphatic vessels (CD31 immunohistochemistry, ×200).

To confirm the presence of lymphatic or venous invasion separately, HE-stained specimens were first evaluated. Then, we prepared sections from the paraffin-blocks corresponding to the respective HE-stained specimen, and D2-40 and CD31 immunostaining together with HE staining was conducted for each case (**Figures 1** and **2**). If there was obvious lymphatic or venous invasion in the HE-stained specimen, then that was recorded accordingly. If there were cancer cells in the lumen lined with endothelial cells positive for the expression of D2-40 or CD31, the decision was based on the concordance of the results of immunohistochemistry with the results of the re-sliced HE-stained specimen. Since D2-40 can stain non-specifically, especially cells other than those of the lymphatic endothelium, including the basal cells (22), we emphasized the comparison with the re-sliced HE-stained specimen. As CD31 immunostaining also faintly stains lymphatic endothelial cells, for cases in which both the expressions of D2-40 and CD31 were positive, we considered the staining intensity of obvious venous endothelial cells on the same section in our decision (**Figure 3**).

#### Perineural Invasion

The presence of perineural invasion was confirmed using the HE-stained specimen, which was routinely prepared for pathological diagnosis. Perineural invasion was defined as complete circumferential or direct invasion of peripheral nerve structures by the adenocarcinoma (23).

#### EPE

EPE is defined as an extension of a tumor into the periprostatic soft tissue (24). This definition has been adopted by the tumor, lymph node, and metastasis staging system for prostate cancer and the ISUP (25). Although EPE in the posterolateral area can be diagnosed when the presence of carcinoma cells is confirmed in the loose connective tissue or perineural spaces of the neurovascular bundles (25), there were no such cases in this study, and cases with firm invasion into the adipose tissue were included as EPE.

#### Surgical Margins

As mentioned above, both the prostate apex and base were examined using the cone method with sagittal sectioning (21). The remaining prostate was entirely cut at approximately 5-mm intervals from the apex to the base, perpendicular to the long axis. All sections were embedded into paraffin and examined. Positive or negative surgical margins were confirmed using the HE-stained specimen, which is prepared routinely for diagnosis. At our institution, blue ink is applied to the prostate’s surface when it is cut. If the cancer cells extend to the ink line at the edge of the prostate tissue, the margin is considered positive.

#### Seminal Vesicle Invasion

Seminal vesicle invasion was detected using histopathological evaluation and defined as a firm invasion of cancer cells into the muscle wall of the seminal vesicle (26). Although EPE and seminal vesicle invasion are similar in that they involve the outside of the prostate, they are considered independent parameters (27) and were evaluated individually in this analysis.

#### IDC-P

**Figure 4:**
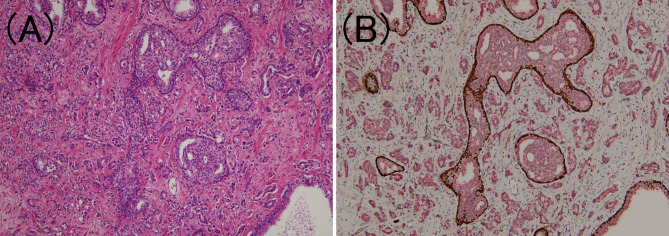
Intraductal carcinoma of the prostate. **(A)** In the lumen of the prostate gland, carcinoma cells identical to those of the surrounding prostate adenocarcinoma components have developed (hematoxylin and eosin staining, ×100). **(B)** Tumor components are stained red owing to P504S immunoreactivity, while the periprostatic gland lumen is stained brown owing to p63 immunoreactivity (PIN4 immunohistochemistry, ×100).

According to the latest ISUP consensus (2), we defined IDC-P as an extension of adenocarcinoma cells into the preexisting prostatic ducts and acini, distending them, with some preservation of the basal cells. Since IDC-P typically arises adjacent to invasive cancer cells and rarely occurs without invasion, we also confirmed the presence of invasive cancer in the surrounding area. Previous studies have reported the following morphological features of IDC-P: expanded growth of carcinoma cells forming large dense cribriform and/or solid structures (28), which were also confirmed in this study. Furthermore, the basal cells are not always confirmed through HE-stained specimens alone (2); therefore, PIN4 immunostaining (combined AMACR (P504S)/34bE12/p63 immunostaining) was performed on one representative section of the specimen to confirm the presence of IDC-P (**Figure 4**). In addition, although controversial, it is commonly considered that IDC-P is not incorporated into GG (29); hence, we exclude it from the GG assessment for IDC-P areas.

#### Tertiary Gleason Pattern 5

**Figure 5:**
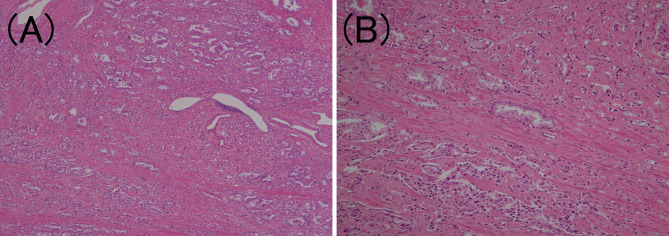
Tertiary Gleason pattern 5 in prostatic adenocarcinoma. **(A)** Most cancer cells correspond to Gleason pattern 4 or 3. (hematoxylin and eosin (HE) staining, ×40). **(B)** The overall picture shows that <5% of the carcinoma cells are solitary or grow in a linear fashion. (HE staining, ×200).

Tertiary Gleason pattern 5 was defined as the percentage of cases with Gleason pattern 5 <5% (30). Cases with tertiary Gleason pattern 5 in GG4 or less were included (**Figure 5**).

#### Biochemical Recurrence After RARP

In line with the American Urological Association (31) and European Association of Urology Guidelines (32) (as well as the Japanese guidelines), BCR was defined by two consecutive rising PSA values >0.2 ng/mL after radical prostatectomy (in this case, the date of the first rise was defined as the date of the BCR). If the serum PSA level did not fall below 0.2 ng/mL after RARP and was 0.2 ng/mL or higher in two successive tests, the date of surgery was assigned as the day of BCR.

#### Lymph Node Metastasis

The presence or absence of lymph node metastasis was confirmed in cases in which lymph node dissection was performed. At our institution, patients who were at a high risk according to the D’Amico classification or those with 7% or higher predicted lymph node metastasis rates according to the Briganti 2012 nomogram (33) underwent lymph node resection.

#### Additional Morphological Analysis

Morphological analysis was conducted in cases where the carcinoma cells had metastasized (cases with EPE, seminal vesicle invasion, or lymph node metastasis were included in the analysis). Specifically, in each case, we identified the Gleason patterns 4 and 5 components of the lesions, which were recognized as high grade. We recorded the presence of the five subtypes each of Gleason patterns 4 and 5 (in this study, papillary/ductal adenocarcinomas were also included as subtypes). These 10 subtypes were based on the ISUP 2014 grading system (20). We recorded subtypes that accounted for at least 10% of the intraprostatic, invasive, and metastatic lesions, respectively. We also recorded the most predominant subtypes. The primary subtype decision was made by YO or AI, who described the specimens. Then, together with the third pathologist (YM), the specimens were reviewed using a multi-viewing biological microscope. In case of disagreement on the subtype, the three pathologists discussed the findings, but if they still failed to agree, the opinion of YM, who had a longer experience of prostate cancer diagnosis, was prioritized.

### Statistical Analyses

For binary variables that could take two values (lymphatic, venous, perineural, seminal vesicle invasion, EPE, positive surgical margins, tertiary Gleason pattern 5, and IDC-P), the Chi-square test was used for statistical analysis of GG and the various parameters. Statistical significance was set at P<0.05. We also measured the adjusted residuals to test for an association between GG and each of the parameters. A value of ±1.96 or higher was considered significant.

Analysis of variance was used to analyze GG and continuous variables (age, preoperative PSA, BMI, and tumor diameter). P<0.05 was considered significant for each group.

Multivariate logistic regression analysis was performed to extract risk factors for BCR and lymph node metastasis in prostate cancer. The dependent variable was the presence or absence of BCR or lymph node metastasis, and the explanatory variables included GG; lymphatic, venous, perineural, and seminal vesicle invasion; EPE; positive surgical margins; tertiary Gleason pattern 5, IDC-P, age, preoperative PSA, BMI, and tumor diameter. These parameters were recorded during the routine pathological diagnosis process. Differences were considered significant at P<0.05. In the present study, all currently available cases were subjected to statistical analyses, but cases involving preoperative hormonal therapy were excluded owing to the impossibility of GG evaluation. In addition, GG1 was also excluded owing to the presence of only two cases.

## Results

**Figure 6:**
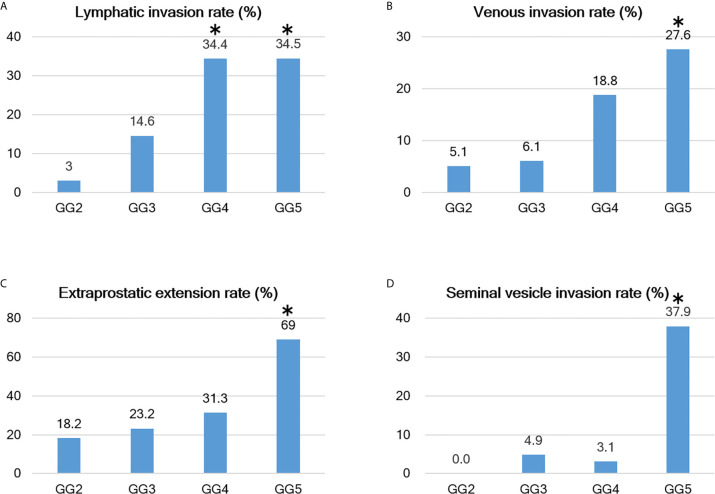
Relationship between Grade group and positive rates of various parameters. **(A–D)** As the Grade group (GG) increases, various evaluation parameters become easier to visualize; however, there are differences between the parameters. For example, the lymphatic invasion rate increases from GG3 and reaches a plateau at GG4, while the venous invasion rate begins to increase at GG4 and is even higher at GG5. Extraprostatic extension (EPE) is detected at a constant frequency starting at GG2 (but becomes extremely high at GG5), and seminal vesicle invasion has a sharp increase in positivity at GG5. The bars with the asterisk symbol (*) in each graph mean that the adjusted residuals are greater than 1.96 in the Chi-square test, indicating that the corresponding values are significantly higher between the groups (e.g., 37.9% for GG5 in seminal vesicle invasion rate is statistically significant).

The rates of the parameters were as follows: lymphatic invasion, 37/299 (12.4%); venous invasion, 25/299 (8.4%); perineural invasion, 280/299 (93.6%); EPE, 89/299 (29.8%); positive surgical margins, 106/299 (35.5%); seminal vesicle invasion, 23/299 (7.7%); tertiary Gleason pattern 5, 33/260 (12.7%); ICD-P, 56/299 (18.7%); and lymph node metastasis, 23/299 (7.7%). These results are summarized in **Table 1** and **Figure 6**. In addition, there were no cases of GS 3 + 5 = 8 or GS 5 + 3 = 8 or cases with microscopic invasion of the bladder neck in this study.

**Table 1:** The mean, standard deviation, or detection rates for the various study parameters.

Total cases	299
Lymphatic invasion rate	37/299 (12.4%)
Venous invasion rate	25/299 (8.4%)
Perineural invasion rate	280/299 (93.6%)
EPE rate	89/299 (29.8%)
Positive surgical margins rate	106/299 (35.5%)
Seminal vesicle invasion rate	23/299 (7.7%)
Tertiary Gleason pattern 5 rate	33/260 (12.7%)
Intraductal carcinoma of the prostate rate	56/299 (18.7%)
Lymph node metastasis rate	23/299 (7.7%)
Age (years, mean ± SD)	67.6 ± 6.4
BMI (mean ± SD)	24 ± 3.1
Preoperative PSA value (ng/ml, mean ± SD)	10.9 ± 13.2
Tumor diameter from surgical specimen (mm, mean ± SD)	24.3 ± 10.2

SD, standard deviation; BMI, body mass index; EPE, extraprostatic extension.

Results of the analysis of the various parameters for all 299 cases.

For all parameters, detailed values, percentages, and adjusted residuals (Chi-square test) for each GG were as follows (**Tables 2** and **3**): Up to GG1 and GG2, there was rarely any lymphatic invasion; however, it was observed in >10% of GG3 cases. In particular, it was confirmed in approximately one-third of the cases for GG4 and GG5. The adjusted residuals for GG4 and GG5 were notably >1.96. GG4 and GG5 had a significant impact on the increased risk of lymphatic invasion. Venous invasion was rarely seen below GG3; contrarily, it was confirmed in approximately one-fifth and one-fourth of the GG4 and GG5 cases, respectively. However, only GG5 had an adjusted residual >1.96, and the overall positivity rate was low compared to that of lymphatic invasion.

**Table 2:** Summary of the evaluation items for each Grade group.

	GG1	GG2	GG3	GG4	GG5
Cases	2	99	82	32	29
Age (years, mean ± SD)	68.5 ± 7.8	67.4 ± 6.2	68.5 ± 5.8	66.2 ± 7.2	68.2 ± 6.2
BMI (mean ± SD)	23.1 ± 0.2	23.8 ± 2.9	23.8 ± 2.9	24.2 ± 3.7	24.7 ± 4.1
Preoperative PSA value (ng/ml, mean ± SD)	12.8 ± 10.6	7.6 ± 4.7	8.4 ± 4.4	9.1 ± 4.8	16.1 ± 24.2
Tumor diameter from surgical specimen (mm, mean ± SD)	10 ± 7.1	23.8 ± 8.2	25.7 ± 9.3	23.2 ± 9.4	31.2 ± 12.8
Lymphatic invasion	0/2 (0%)	3/99 (3.0%)	12/82 (14.6%)	11/32 (34.4%)	10/29 (34.5%)
Venous invasion rate	0/2 (0%)	5/99 (5.1%)	5/82 (6.1%)	6/32 (18.8%)	8/29 (27.6%)
Perineural invasion rate	0/2 (0%)	93/99 (93.9%)	82/82 (100%)	32/32 (100%)	29/29 (100%)
EPE rate	0/2 (0%)	18/99 (18.2%)	19/82 (23.2%)	10/32 (31.3%)	20/29 (69%)
Positive surgical margins rate	0/2 (0%)	29/99 (29.3%)	29/82 (35.4%)	10/32 (31.3%)	20/29 (69%)
Seminal vesicle invasion rate	0/2 (0%)	0/99 (0%)	4/82 (4.9%)	1/32 (3.1%)	11/29 (37.9%)
Tertiary Gleason pattern 5 rate	0/2 (0%)	8/99 (8.1%)	21/82 (25.6%)	4/32 (12.5%)	none
IDC-P rate	0/2 (0%)	4/99 (4.0%)	15/82 (18.3%)	11/32 (34.4%)	17/29 (58.6%)
Lymph node metastasis rate (All patients except for those who underwent preoperative hormonal therapy)	0/2 (0%)	2/99 (2.0%)	4/82 (4.9%)	1/32 (3.1%)	9/29 (31.0%)
Lymph node metastasis rate (Only cases in which lymph node dissection was conducted)	None	2/31 (6.5%)	4/50 (8.0%)	1/26 (3.8%)	9/27 (33.3%)

GG, Grade group; SD, standard deviation; BMI, body mass index; EPE, extraprostatic extension; IDC-P, Intraductal carcinoma of the prostate.

Summary of the mean, standard deviation, or detection rate of the various parameters for each Grade group.

**Table 3:** Results from the adjusted residuals, in which various parameters based on the Chi-square test were detected, for each Grade group.

	GG2	GG3	GG4	GG5
Lymphatic invasion	-4.3	0	3.3	3.1
Venous invasion rate	-2.1	-1.4	1.8	3.4
Perineural invasion rate	-3.0	1.8	1	0.9
EPE	-2.8	-1.1	0.5	5.3
Positive surgical margin	-1.9	-0.2	-0.7	3.9
Seminal vesicle invasion	-3.5	-0.8	-0.9	7.2
Tertiary Gleason pattern 5	-2.8	-3.2	-0.5	None
IDC-P	-5.1	-0.3	2.3	5.8
Lymph node metastasis rate (Only cases in which lymph node dissection was conducted)	-1.1	-1.1	-1.4	3.8

GG, Grade group; EPE, extraprostatic extension; IDC-P, Intraductal carcinoma of the prostate.

Based on the Chi-square test, the adjusted residuals for the various parameters between the groups were evaluated; ± 1.96 was used as a criterion for the presence of a significant difference, and the detection rate was considered significantly high if it was >1.96 and significantly low if it was ≤1.96. GG1 was excluded from the analysis owing to the excessively limited number of cases.

Most of the cases were positive for perineural invasion. EPE occurred at a constant frequency of approximately one-fifth to one-fourth of the cases in GG2 to GG4, though its occurrence was significantly lower in GG2. By contrast, GG5 exhibited a significantly higher rate of positivity than the other groups (slightly more than two-thirds were confirmed in GG5).

Positive surgical margins were found at a constant rate but were significantly higher in GG5. The rates in 2019 and 2020 were different, at 49/120 (40.8%) and 39/124 (31.5%), respectively, but the difference was not significant (Chi-square test, P-value=0.152).

Seminal vesicle invasion was the most strongly affected parameter in GG5 and was significantly higher in GG5. The invasion was found in approximately one-third of the GG5 cases but less than 5% in GG4 or below cases.

The incidence of tertiary Gleason pattern 5 was significantly higher in GG3 than in GG2. In GG4, the rate was relatively lower than that in GG3.

The incidence of IDC-P represented approximately one-fifth of cases with GG3. The adjusted residuals for GG4 and GG5 were notably >1.96. GG4 and GG5 had a significant impact on the increased risk of IDC-P. In addition, there were no cases of comedonecrosis with IDC-P in this study.

Regarding the lymph node metastasis rate in patients who underwent lymph node dissection up to GG4, it was <10% (some difference was present; however, it was not significant). Contrastingly, GG5 exhibited lymph node metastasis in approximately one-third of cases, which was significantly higher than the findings from other GG groups.

The continuous variables PSA levels and tumor diameter were significantly higher in GG5 than in other groups. In contrast, there were no significant differences in any variables between GG2 and GG4.

In this study, postoperative BCR was observed in 31/242 (12.8%) cases; cases with preoperative hormone therapy were excluded from this analysis. At our hospital, serum PSA levels are measured at least twice for each radical prostatectomy to decide the treatment and follow-up strategy. Therefore, patients who received additional treatment before being diagnosed as BCR were not included in this study. GG2, GG3, GG4, and GG5 accounted for four, seven, seven, and 13 cases, respectively. The average time to diagnosis of BCR was 111.8 days (range: 0 to 543 days); 19/31 (61.3%) cases never had PSA<0.2 ng/mL, postoperatively, and BCR for them was assigned to the day of surgery. We also examined the incidence of BCR in cases with EPE, seminal vesicle invasion, or lymph node metastasis, which were 17/67 (25.4%), 8/16 (50%), and 9/16 (56.3%), respectively. Our morphological analysis showed that in each of the analyses, the most prominent subtypes of intraprostatic lesions were small and large fused glands, but there were differences in their distribution (**Tables 4**–**6**). We conducted multivariate logistic regression analysis to extract independent risk factors for BCR in this study and found that GG and tumor diameter were significant risk factors for BCR. Lymph node metastasis was not a significant risk factor, though it tended toward significance. The odds ratios for BCR with respect to GG and tumor diameter were 2.253 (95% confidence interval: 1.297–3.912; P=0.004) and 1.074 (95% confidence interval: 1.011–1.142; P=0.022), respectively (**Table 7**).

**Table 4:** Association between morphological characteristics of intraprostatic and invasive lesions in cases with EPE.

	Predominant morphological variant (intraprostatic lesion)	Cases with BCR (intraprostatic lesion)	Cases with BCR (invasion lesion)	Cases without BCR (intraprostatic lesion)	Cases without BCR (invasive lesion)
Ill formed	5/67 (7.5%)	9/17 (52.9%)	8/17 (47.1%)	28/50 (56%)	13/50 (26%)
Small and large fused	32/67 (47.8%)	11/17 (64.7%)	8/17 (47.1%)	40/50 (80%)	40/50 (80%)
Glomeruloid	4/67 (6%)	4/17 (23.5%)	0/17 (0%)	17/50 (34%)	8/50 (16%)
Cribriform	15/67 (22.4%)	10/17 (58.8%)	5/17 (29.4%)	26/50 52%)	9/50 (18%)
Papillary	8/67 (11.9%)	3/17 (17.6%)	1/17 (5.9%)	16/50 (32%)	0/50 (0%)
Single cell	0/67 (0%)	9/17 (52.9%)	3/17 (17.6%)	13/50 (26%)	2/50 (4%)
Single file	0/67 (0%)	8/17 (47.1%)	3/17 (17.6%)	12/50 (24%)	1/50 (2%)
Cribriform with comedonecrosis	0/67 (0%)	3/17 (17.6%)	0/17 (0%)	9/50 (18%)	1/50 (2%)
Pseudorosetting	0/67 (0%)	0/17 (0%)	0/17 (0%)	0/50 (0%)	0/50 (0%)
Solid	3/67 (4.5%)	6/17 (35.3%)	1/17 (5.9%)	2/50 (4%)	1/50 (2%)

EPE, extraprostatic extension; BCR, biochemical recurrence.

The subtypes of Gleason patterns 4 and 5 in cases with EPE were examined both in intraprostatic and invasive lesions, respectively. Overall, the “small and large fused glands” subtype was the predominant subtype in the intraprostatic lesions. In addition, the Gleason pattern 5 component was more likely to be observed in cases with BCR than in cases without BCR in both intraprostatic and invasive lesions.

**Table 5:** Association between morphological characteristics of intraprostatic and invasive lesions in cases with SVI.

	Predominant morphological variant (intraprostatic lesion)	Cases with BCR (intraprostatic lesion)	Cases with BCR (invasion lesion)	Cases without BCR (intraprostatic lesion)	Cases without BCR (invasive lesion)
Ill formed	1/16 (6.3%)	4/8 (50%)	5/8 (62.5%)	7/8 (87.5%)	5/8 (62.5%)
Small and large fused	7/16 (43.8%)	6/8 (75%)	0/8 (0%)	5/8 (62.5%)	5/8 (62.5%)
Glomeruloid	1/16 (6.3%)	0/8 (0%)	1/8 (12.5%)	4/8 (50%)	2/8 (25%)
Cribriform	4/16 (25%)	4/8 (50%)	0/8 (0%)	6/8 (75.0%)	1/8 (12.5%)
Papillary	0/16 (0%)	0/8 (0%)	5/8 (62.5%)	1/8 (12.5%)	0/8 (0%)
Single cell	0/16 (0%)	6/8 (75%)	4/8 (50%)	6/8 (75%)	2/8 (25.0%)
Single file	1/16 (6.3%)	6/8 (75%)	0/8 (0%)	6/8 (75%)	3/8 (37.5%)
Cribriform with comedonecrosis	0/16 (0%)	1/8 (12.5%)	0/8 (0%)	1/8 (12.5%)	0/8 (0%)
Pseudorosetting	0/16 (0%)	0/8 (0%)	2/8 (12.5%)	0/8 (0%)	0/8 (0%)
Solid	2/16 (12.5%)	3/8 (37.5%)	1/8 (12.5%)	2/8 (25%)	1/8 (12.5%)

SVI, seminal vesicle invasion; BCR, biochemical recurrence.

I In cases with seminal vesicle invasion, the subtypes of Gleason pattern 4 and 5 were examined in both intraprostatic and invasive lesions, respectively. Overall, the “small and large fused glands” subtype was the predominant subtype in the intraprostatic lesions. In addition, the Gleason pattern 5 component was more likely to be observed in both intraprostatic and invasive lesions, regardless of the presence of BCR.

**Table 6:** Association between morphological characteristics of intraprostatic and metastatic lesions in cases with lymph node metastasis.

	Predominant morphological variant (intraprostatic lesion)	Cases with BCR (intraprostatic lesion)		Cases with BCR (metastatic lesion)	Cases without BCR (intraprostatic lesion)	Cases without BCR (metastatic lesion)
Ill formed	2/16 (12.5%)	5/9 (55.6%)	2/9 (22.2%)		5/7 (71.4%)	4/7 (57.1%)
Small and large fused	5/16 (31.3%)	6/9 (66.7%)	1/9 (11.1%)		5/7 (71.4%)	0/7 (0%)
Glomeruloid	0/16 (0%)	1/9 (11.1%)	7/9 (77.8%)		1/7 (14.3%)	4/7 (42.9%)
Cribriform	3/16 (18.8%)	4/9 (44.4%)	2/9 (22.2%)		6/7 (85.7%)	0/7 (0%)
Papillary	3/16 (18.8%)	2/9 (22.2%)	0/9 (0%)		3/7 (42.9%)	0/7 (0%)
Single cell	0/16 (0%)	5/9 (55.6%)	0/9 (0%)		3/7 (42.9%)	0/7 (0%)
Single file	1/16 (6.3%)	5/9 (55.6%)	1/9 (11.1%)		2/7 (28.6%)	3/7 (42.9%)
Cribriform with comedonecrosis	0/16 (0%)	2/9 (22.2%)	1/9 (11.1%)		1/7 (14.3%)	3/7 (28.6%)
Pseudorosetting	0/16 (0%)	0/9 (0%)	3/9 (33.3%)		0/7 (0%)	2/7 (28.6%)
Solid	2/16 (12.5%)	3/9 (33.3%)	1/9 (11.1%)		2/7 (28.6%)	1/7 (14.3%)

BCR, biochemical recurrence.

In cases with lymph node metastasis, the subtypes of Gleason pattern 4 and 5 were examined in both intraprostatic and metastatic lesions, respectively. Though the “small and large fused glands” subtype was slightly predominant, various subtypes tended to be identified in the intraprostatic lesions. In addition, lymph node metastatic lesions tended to congregate to some extent rather than being solitary, while the “Pseudorosetting” formation was observed at a certain frequency.

**Table 7:** Multivariate logistic regression analysis of biochemical recurrence.

Variable	OR (95% CI)	*P*-value
GG	2.253 (1.297–3.912)	0.004
Tumor diameter	1.074 (1.011–1.142)	0.022
Lymph node metastasis	4.074 (0.857–19.358)	0.077

CI, confidence interval; OR, odds ratio; GG, Grade group.

In this multivariate analysis, the GG and tumor diameter were significant independent risk factors for biochemical recurrence. Though it tended to be significant, lymph node metastasis was not a significant factor. This statistical analysis only included cases in which lymph node dissection was conducted.

To explore the risk factors for lymph node metastasis, we performed a multivariate logistic regression analysis. The results established that the presence of lymphatic invasion, EPE, and seminal vesicle invasion were independent risk factors for lymph node metastasis. The odds ratios for the presence of lymphatic invasion, EPE, and seminal vesicle invasion were 7.425 (95% confidence interval: 1.688–22.583; P=0.004), 4.391 (95% confidence interval: 1.037–18.589; P=0.044), and 5.755 (95% confidence interval: 1.308–25.316; P=0.021), respectively (**Table 8**).

**Table 8:** Multivariate logistic regression analysis of lymph node metastasis.

Variable	OR (95% CI)	*P*-value
Lymphatic invasion	7.425 (1.688–22.583)	0.004
EPE	4.391 (1.037–18.589)	0.044
Seminal vesicle invasion	5.755 (1.308–25.316)	0.021

CI, confidence interval; OR, odds ratio; EPE, extraprostatic extension.

In this multivariate analysis, lymphatic invasion, EPE, and seminal vesicle invasion were significant independent risk factors for lymph node metastasis. This statistical analysis only included cases in which lymph node dissection was conducted.

As the analysis found three independent risk factors for lymph node metastasis (lymphatic invasion, EPE, and seminal vesicle invasion), we investigated the relationship between the presence of EPE, seminal vesicle invasion, and lymphatic invasion rate using the Chi-square test. The results verified that there was no significant difference between the lymphatic invasion rate and EPE in patients with EPE when compared with those without EPE. In contrast, more than half of the patients with seminal vesicle invasion had lymphatic invasion, while the lymphatic invasion rate was significantly lower in patients who did not have seminal vesicle invasion (Chi-square test, P< 0.001, **Table 9**).

**Table 9:** Relationship between lymphatic invasion, extraprostatic extension, and seminal vesicle invasion.

Variable	Lymphatic invasion rate	*P*-value (Chi-square test)
Cases with extraprostatic extension	19.4% (13/67)	0.208
Cases without extraprostatic extension	13% (23/177)	
Cases with seminal vesicle invasion	56.3% (9/16)	<0.001
Cases without seminal vesicle invasion	11.8% (27/228)	

The presence of extraprostatic extension did not differ significantly from the lymphatic invasion rate. In contrast, patients with seminal vesicle invasion had a significantly higher lymphatic invasion rate.

## Discussion

In this study, we analyzed the risk factors for BCR and lymph node metastasis in patients who underwent RARP using detailed morphological, immunohistochemical, and statistical analyses of surgical specimens. Furthermore, we clarified the relationship between GG and the assessment of parameters. Though GG is the best known indicator for identifying malignant potential (30), few studies have investigated the relationship between GG and the various clinicopathological parameters that were precisely evaluated through immunohistochemistry for lymphatic invasion, venous invasion, and IDC-P identification. In addition, all risk factors for lymph node metastasis in patients who have undergone RARP have not yet been elucidated (17). Thus, herein, we discuss BCR and lymph node metastasis as prognostic factors in prostate cancer.

Approximately 10% of the cases in the present study were diagnosed as BCR, but for more than half of them, the event was assigned to the day of surgery. This can be partly explained by the short observation period of this study. According to the multivariate analysis, GG and tumor diameter were independent significant factors for BCR, while lymph node metastasis was not a significant factor in this study, even though its P-value tended toward significance. Many previous studies demonstrated lymph node metastasis as a risk factor for BCR instead of GG and tumor diameter (34–37). Detectable serum PSA values after prostatectomy should be closely associated with the presence of residual tumor (38) and intraprostatic incision into benign glands (39). In this study, the BCR was assessed for a short period of time, and hence, further follow-up studies are required to clarify the factors that influence each other.

Considering the overall short follow-up period of this study, we would like to raise a possibility that GG and tumor diameter may have implications as risk factors for very early BCR. In addition, extraprostatic involvement including EPE, seminal vesicle invasion, and lymph node metastasis was not a significant factor for BCR in the multivariate analysis in this study.

We also added the morphological analysis referring to the ISUP 2014 grading system (19, 20). Specifically, in each case, we identified the Gleason patterns 4 and 5 components of the lesions, which are recognized as high grade (3, 40). Our morphological analysis showed that among the cases with EPE, those with BCR tended to have a component of GG5 in both intraprostatic and invasive lesions. Furthermore, GG5 was more likely to be identified in cases with seminal vesicle invasion regardless of BCR occurrence (in both intraprostatic and invasive lesions). In comparison, various subtypes of histology were found in the main lesions of the prostate in cases with lymph node metastasis. It was also found that isolated carcinoma cells were not evident in the metastatic foci in the lymph nodes; thus, showing some degree of aggregation. Expression of paxillin, reported in prostate cancer (41) and involved in cancer cell aggregation (42), could be implicated to this observation; further studies are needed to clarify this relationship. The glomeruloid pattern was relatively rare in the present study. We also examined the most predominant GG4 and GG5 histological subtypes in prostatic lesions using cases with EPE, seminal vesicle invasion, or lymph node metastasis. In all analyses, the cribriform pattern, which has been reported (43–45) to be a poor prognostic factor, was the second common subtype after the small and large fused glands subtype. Thus, a re-evaluation of BCR with a longer observation period is required.

Meanwhile, in this study, 23 (12.8%) of the 179 patients who underwent lymph node dissection had lymph node metastasis. The Chi-square test demonstrated no significant difference between GG2 and GG4; however, lymph node metastasis was found in about one-third of the GG5 cases and this finding was significantly higher than that for the other groups. This result shows that compared with other groups, GG5 exhibited a significantly higher risk of lymph node involvement. Interestingly, GG was not an independent risk factor for lymph node metastasis in the multivariate analysis in this study. Though GG is considered a risk factor for lymph node metastasis (11), the results from our multivariate analysis were inconsistent with those of previous reports (8, 30, 46). One reason for this could be that there were few lymph nodes. In particular, only one GG4 case had lymph node metastasis, which may have affected the results. To mitigate this problem, additional case detail collection is required. In this article, we would like to further discuss the results of the multivariate analysis using cases in which lymph node dissection was conducted. In the statistical analysis, lymphatic invasion, EPE, and seminal vesicle invasion were independent risk factors for lymph node metastasis. At our institution, to avoid prolonged operative times, damage to blood vessels and nerves, and postoperative lymphatic circulation disturbance, lymph node dissection is conducted if the patient is at high risk according to the D’Amico classification or if the predicted Briganti 2012 lymph node metastasis rate is >7%. Because the criteria for lymph node dissection included factors other than GG (PSA, preoperative staging by radiologists, and core-positive rates on preoperative biopsy), the influence of other factors may have been stronger in patients with relatively low GG. Consequently, we propose that GG may not have been an independent risk factor for patients who underwent lymph node dissection at our institution. From another perspective, the three independent risk factors for lymph node metastasis identified in the present multivariate analysis were assumed to have a strong influence on lymph node metastasis in addition to GG. Therefore, we would like to discuss these risk factors further.

Lymphatic vessels are the pathways to lymph nodes, and lymphatic invasion is a risk factor for lymph node metastasis (11, 47). It should be noted that venous invasion was not a risk factor for lymph node metastasis in our study. Considering that only lymphatic invasion is an independent risk factor for lymph node metastasis (15), lymphatic and venous invasion should be assessed separately rather than combined into the category of lymphovascular invasion. However, venous invasion is generally considered a risk factor for distant metastasis, and previous studies that evaluated lymphatic and venous invasion separately (but not in the prostate) reported that venous invasion is a risk factor for distant metastasis (48, 49). Unfortunately, studies analyzing only venous invasion in prostate cancer are scarce, and further long-term studies are required to elucidate its significance.

It is worth mentioning that seminal vesicle invasion was also an independent risk factor for lymph node metastasis. This could be owing to the anatomy of the seminal vesicle or its proximity to the prostate. The area surrounding the seminal vesicle is rich with lymphatic vessels (4.1 mm^2^) (50). In contrast, the lymphatic vessel density in a normal prostate is approximately 1.58 mm^2^ (51). In fact, the lymphatic invasion rate is significantly higher in patients with seminal vesicle invasion than in those without seminal vesicle invasion. In this study, it was approximately five times greater (**Table 5**). It is possible that cancer cells that invade the seminal vesicles may be more directly related to the lymphatic pathway owing to the high density of lymphatic vessels in the area.

EPE was also an independent risk factor for lymph node metastasis. However, we did not observe a significant relationship between EPE and lymphatic invasion in this study. Though we precisely evaluated lymphatic invasion with HE staining, supported by D2-40 immunohistochemistry, there could have been lymphatic invasion that was not detectable microscopically (52). In addition, cases with EPE had approximately two times the total incidence of BCR even in the short period of time in this study. However, multivariate analysis of this study showed that EPE was not an independent significant factor for BCR. To better understand these observations, further analysis, preferably molecular analysis, is required.

Further discussion is warranted regarding the relationship between GG and other parameters. Our analysis established that, in general, as the GG increased, the positive rates of various pathological evaluation parameters increased. However, a detailed examination of the mean values and detection rates of the various evaluation parameters for each GG confirmed the differences between the parameters. Thus, we would like to discuss the various parameters in terms of the statistical analysis results. At first, in addition to the routine examination of HE-stained specimens, we conducted an additional re-evaluation of HE staining and immunohistochemistry with D2-40 or CD31 for representative sections and precisely evaluated the vessel invasions. D2-40 is reported to also stain cells other than those of the lymphatic endothelium (22) and CD31 also faintly stains lymphatic vessels; therefore, it is essential to ensure that both D2-40 and CD31 immunohistochemistry are conducted with HE staining. Our precise differential evaluation of lymphatic and venous invasions confirmed that lymphatic invasion was positively associated with lymph node metastasis and extraprostatic extension. Perineural invasion was positive in most cases, but its value for evaluation is questionable. Semi-quantitative methods of evaluation, such as infiltration severity could improve the value, but further verification is required. From GG2 to GG4, positive surgical margins were observed in about one-third of cases, and in GG5, positive surgical margins were observed in more than two-thirds of cases. Thus, the positive surgical margins in GG5 were significantly higher than in cases up to GG4. As a matter of concern, the rate of positive surgical margins was lower in 2020 than in 2019, although not significantly different. Therefore, we must follow the progress carefully, including the rate of positive surgical margins in the future. Seminal vesicle invasion was rarely observed in GG4 and below, but similar to the findings for EPE, the frequency was significantly elevated in GG5 cases. Cases of GG5 seemed to be an apparently malignant disease compared with cases of GG4. The incidence of tertiary Gleason pattern 5 was significantly higher in GG3 than in GG2, while the incidence in GG4 was relatively lower than that in GG3. When the amount of pattern 5 exceeds 5%, the pattern 5 was not considered as the tertiary component but included in the grade. The higher grade tumors tend to have larger amounts of pattern 5 >5%, which might be the reason for the relatively low incidence of tertiary Gleason pattern 5 in GG4 cases in this study. IDC-P is a poor prognostic factor in prostate cancer (8), and it was observed at a frequency of about one-fifth even in GG3 cases. Therefore, in cases of GG3 and above, immunohistochemical analysis using PIN4 or other methods should actively be performed when there is a suspicious site in the routine diagnosis using HE staining. The continuous variables, PSA and tumor diameter, were significantly higher in GG5 than in other groups. In contrast, there were no significant differences in any variables up to GG4. This might be because the present statistical analysis was limited to patients who were judged as operable. Nevertheless, we once more wish to state the limitations of this study. The study includes cases of radical prostatectomy, which were performed after January 2019. Therefore, the maximum follow-up period is approximately 2.5 years. The short follow-up duration is a limitation of this study. Further follow-up is required for analysis of biochemical and clinical recurrence, metastasis, and prognosis. Furthermore, accumulation of morphological analysis is also necessary.

In conclusion, this study elucidated the risk factors for BCR and lymph node metastasis in　patients who underwent RARP using detailed morphological and immunohistochemical analyses, and found that the independent risk factors for BCR were GG and tumor diameter, while the independent risk factors for lymph node metastasis were lymphatic and seminal vesicle invasion and the presence of EPE. Additionally, the study successfully characterized the status of various parameters for each GG in prostate cancer. As GG increased, various parameters could be easily visualized. Compared with other groups, the GG5 group exhibited higher frequencies of various parameters for disease progression. Furthermore, these results have identified the assessment parameters for each GG as well as the differences in the biological malignancy of GG5. Further investigation of the differences between GG5 and other groups regarding various aspects (including morphological analyses) may provide the basis for delineating some of the malignant features of prostate cancer.

## Data Availability Statement

The raw data supporting the conclusions of this article will be made available by the authors, without undue reservation.

## Ethics Statement

The studies involving human participants were reviewed and approved by Ethics Review Committee of the Kanagawa Cancer Center (Approval Number: 2019-36). The patients/participants provided their written informed consent to participate in this study.

## Author Contributions

YO diagnosed RARP specimens, collected the parameters, performed the statistical analysis, and wrote the manuscript. SS diagnosed RARP specimens and discussed the GG with YO and YM. KO was the primary operator of the RARP procedure, mentored other urologists, and provided YO with the clinical information in this study. YY provided information on preoperative imaging findings to YO. TS actively performed the RARP surgery together with KO and provided YO with clinical information in this study. AI confirmed the presence of BCR, date of diagnosis of the BCR, and serum PSA level from the medical records during the manuscript revision. AI also reviewed the specimens with YO and participated in the morphological examinations. EY, MS, KW, and TY diagnosed some of the preoperative biopsies and provided that information to YO. In addition, they revise this article from the perspective of a pathologist. TK, as head of the urologist department, provided clinical information to YO and revise parts of the manuscript. YM reviewed and reassessed the specimens and revised the manuscript as the senior pathologist. All authors contributed to the article and approved the submitted version.

## Funding

This work was supported by JSPS KAKENHI (grant number: JP17K08713 to YO, 20K09422 to SS, 20K16210 to KW, 18K15111 to MS, 20K09093 to YM) from the Ministry of Education, Culture, Sports, Science, and Technology of Japan and by Kanagawa Cancer Center and Research Institute/Kanagawa Prefectural Hospital Organization (grant number: 2020-4/2021-1 to YO).

## Conflict of Interest

The authors declare that the research was conducted in the absence of any commercial or financial relationships that could be construed as a potential conflict of interest.

## Publisher’s Note

All claims expressed in this article are solely those of the authors and do not necessarily represent those of their affiliated organizations, or those of the publisher, the editors and the reviewers. Any product that may be evaluated in this article, or claim that may be made by its manufacturer, is not guaranteed or endorsed by the publisher.

## Abbreviations

PSA, prostate-specific antigen; BMI, body mass index; GG, Grade group; GS, Gleason Score; EPE, extraprostatic extension; BCR, biochemical recurrence; IDC-P, intraductal carcinoma of the prostate gland; RARP, robot-assisted radical prostatectomy; HE, hematoxylin and eosin.
